# Hepatic Artery Thrombosis: A Rare Complication of Microwave Ablation in Hepatocelluar Carcinoma

**DOI:** 10.7759/cureus.6811

**Published:** 2020-01-29

**Authors:** Amreet K Aujla, Leon D Averbukh, Guneetinder Gill, Colin Swales

**Affiliations:** 1 Internal Medicine, University of Connecticut Health Center, Farmington, USA; 2 Internal Medicine, Baba Farid University of Health and Sciences, Dayanand Medical Hospital and College, Ludhiana, IND; 3 Gastroenterology, University of Connecticut, Hartford, USA

**Keywords:** hepatocellular carcinoma, microwave ablation, portal vein thrombosis

## Abstract

Microwave ablation (MWA) has become a popular therapeutic technique in hepatocellular carcinoma (HCC) alongside cryoablation, radiofrequency ablation, and liver resection/transplantation in patients with limited tumor burden. Generally well tolerated, and not as invasive as surgery, the technique results in low mortality and complication rates. We report the exceedingly rare complication of hepatic artery thrombosis with subsequent fatal ischemia of the left hepatic lobe in a 64-year-old female with cirrhosis and HCC who underwent MWA.

## Introduction

Microwave ablation (MWA) has become a popular therapeutic technique alongside cryoablation and radiofrequency ablation in patients with limited tumor burden hepatocellular carcinoma (HCC). The non-surgical technique benefits from good patient tolerability and low rates of serious complications. We describe the case of a 64-year-old female with compensated cirrhosis and HCC who, after receiving MWA to liver segment IV, experienced a very rare and fatal complication: left lobar hepatic ischemia secondary to hepatic artery thrombosis.

## Case presentation

**Figure 1:**
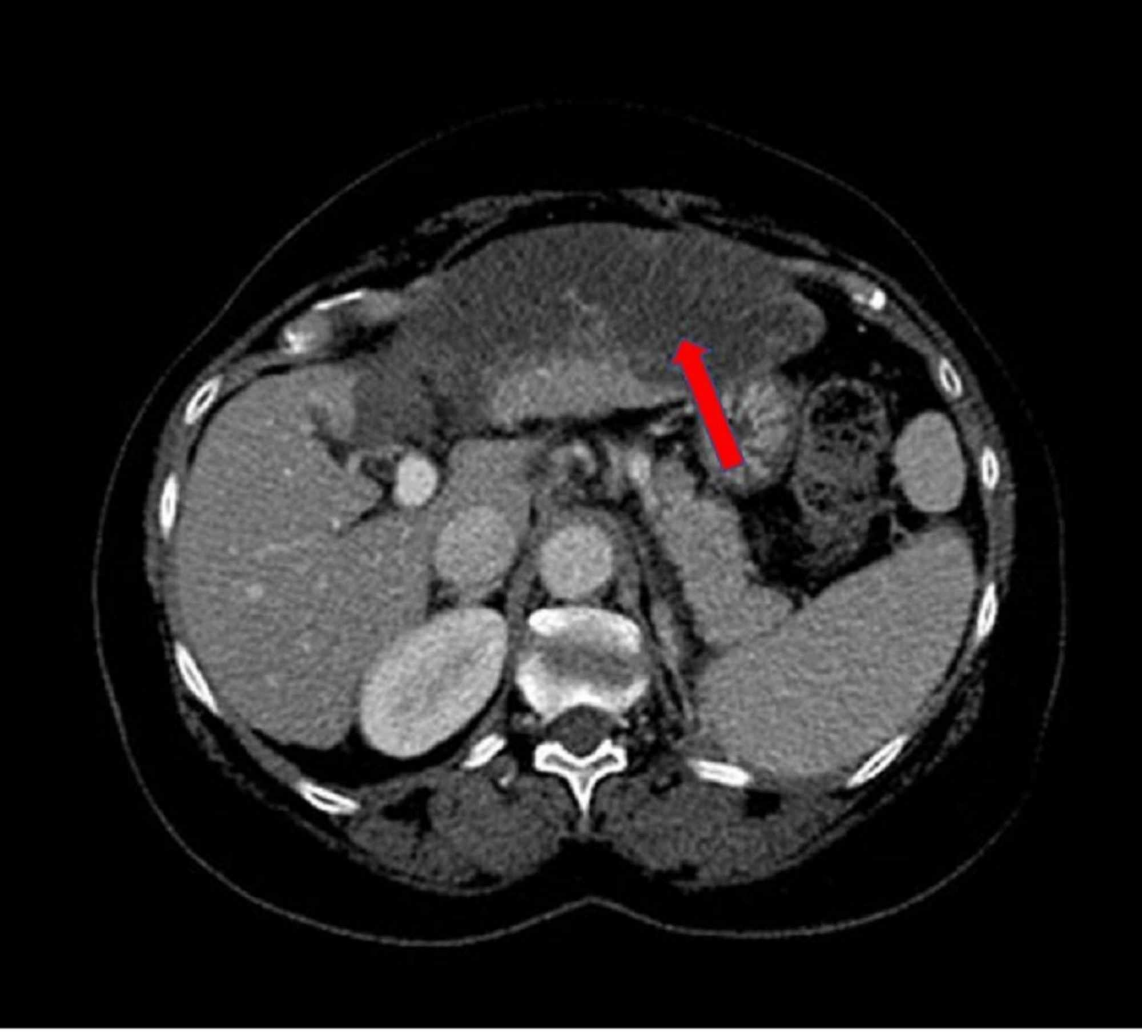
CT angiogram of the abdomen revealing thrombosis of the left branch of the hepatic artery with hepatic infarct (red arrow).

A 64-year-old female presented to the hospital with the chief complaint of right upper quadrant pain, multiple episodes of non-bilious emesis, and anorexia since undergoing a computer tomography (CT)-guided microwave ablation of an HCC tumor in liver segment IV the day before. The patient’s past medical history was significant for untreated hepatitis C, compensated liver cirrhosis, and HCC stage IA diagnosed in 2017 with a single lesion measuring 1.5 x 2.0 cm in diameter. At the time of presentation, the patient was hypotensive to 81/52 mmHg and tachycardia to 118 beats per minute. Physical examination was significant for scleral icterus, abdominal distension, right upper quadrant tenderness and hypoactive bowel sounds. Laboratory studies demonstrated an aspartate aminotransferase of 2,467 U/L, an alanine aminotransferase of 688 U/L, an alkaline phosphatase of 355 U/L, a total bilirubin of 4.5 mg/dL, and an international normalized ratio of 1.1. CT angiogram of the abdomen revealed thrombosis of left branch of the hepatic artery with hepatic infarct (Figure 1). Upon hospitalization, the patient experienced hypoxic respiratory failure, hepatic encephalopathy, transfusion dependent anemia, and hepatorenal syndrome. Her Model for End-Stage Liver Disease-Na scores during admission escalated from 11 to 49 precluding emergent liver transplant and regrettably, the patient expired from multi-organ failure.

## Discussion

MWA has become a popular therapeutic technique in isolated HCC alongside cryoablation, radiofrequency ablation, and liver resection/ transplantation [1]. MWA uses electromagnetic waves between 300 MHz and 300 GHz to produce tissue heating via oscillations of polar molecules that in turn cause frictional heating. Ultimately, this heating process causes tissue necrosis within solid tumors. MWA has a low mortality rate of 0% to 0.4% and a major complication rate of 2.6% to 4.6% [2]. The technique is indicated for curative purposes in patients who meet the Milan criteria (one tumor nodule less than 5 cm or three tumor nodules less than 3 cm, no portal vein cancerous thrombus, and no extrahepatic spread of disease) [3]. Major complications and causes of death associated with MWA include intraperitoneal bleeding, portal vein thrombosis, intrahepatic hematomas, bile leak, biloma, bile duct injury, liver dysfunction, liver abscess, intestinal perforation, diaphragmatic hernia, hemothorax, intractable pleural effusion, and tumor implantation [4]. On review of literature, the incidence of ischemic injury in MWA is less than 1% [5]. A multi-center study which evaluated for complications of MWA for liver tumors in a cohort of 736 patients showed only one patient who experienced hepatic infarction during the procedure, with successful subsequent medical management [6]. Importantly, there was no correlation found between incidence of major complications post MWA and the time of exposure, the presence of chronic liver disease, tumor type, site, or size [5]. The only other reported case in literature of hepatic ischemia was the result of emergent post MWA vessel embolization due to hepatic hemorrhage in the setting of antenna malposition [7].

Although our patient met the Milan criteria for liver transplantation in the setting of HCC and cirrhosis with a single tumor lesion of 1.5 x 2.0 cm, she chose to undergo MWA due to its minimal invasiveness, excellent tolerability and safety profile, proven efficacy in local disease control, virtually unlimited repeatability, and cost-effectiveness [5]. Unfortunately, MWA of her lesion in hepatic segment IV caused thrombosis of the segment’s arterial feeder vessel, the left branch of the hepatic artery, and resulted in left lobar liver ischemia. While thrombosis of the portal vein is a known complication of MWA, the hepatic arteries are generally spared due to a combination of their large diameter (over 3 mm) and high flow state [8].

This work has been presented as a poster at the Annual Scientific Meeting of the American College of Gastroenterology, Philadelphia, PA, October 7, 2018 (Aujla AK, Shah N, Sharma T, Swales C. P0647-Hepatic Artery Thrombosis: A Rare Complication of Microwave Ablation in Hepatocellular Carcinoma, https://eventscribe.com/2018/ACG/ajaxcalls/PosterInfo.asp?PosterID=160194&efp=RFNSWFFHSFY2NDI0&rnd=0.8721181)

## Conclusions

Although MWA is generally regarded as a safe procedure, it is important to not only know and understand its common complications, but also the potentially life threatening hepatic segment dependent ones. While post procedural thrombosis of the portal vein and narrow lumen, low flow hepatic vessels has been well documented with MWA therapy, on very rare occasions, larger, high flow vessels such as the hepatic arteries may also become involved with catastrophic consequences. 
